# Digital image analysis of endoscopic ultrasonography is helpful in diagnosing gastric mesenchymal tumors

**DOI:** 10.1186/1471-230X-14-7

**Published:** 2014-01-08

**Authors:** Gwang Ha Kim, Kwang Baek Kim, Seung Hyun Lee, Hye Kyung Jeon, Do Youn Park, Tae Yong Jeon, Dae Hwan Kim, Geun Am Song

**Affiliations:** 1 Department of Internal Medicine, Pusan National University School of Medicine and Biomedical Research Institute, Pusan National University Hospital, Busan, Korea; 2 Division of Computer Engineering, Silla University, Busan, Korea; 3 Department of Pathology, Pusan National University School of Medicine, Busan, Korea; 4 Department of Surgery, Pusan National University School of Medicine, Busan, Korea

**Keywords:** Stomach, Endoscopic ultrasonography, Mesenchymal tumor, Image analysis

## Abstract

**Background:**

Endoscopic ultrasonography (EUS) is a valuable imaging tool for evaluating subepithelial lesions in the stomach. However, there are few studies on differentiation between gastrointestinal stromal tumors (GISTs) and benign mesenchymal tumors, such as leiomyoma or schwannoma, with the use of EUS. In addition, there are limitations in the analysis of the characteristic features of such tumors due to poor interobserver agreement as a result of subjective interpretation of EUS images. Therefore, the aim of this study was to evaluate the role of digital image analysis in distinguishing the features of GISTs from those of benign mesenchymal tumors on EUS.

**Methods:**

We enrolled 65 patients with histopathologically proven gastric GIST, leiomyoma or schwannoma on surgically resected specimens who underwent EUS examination at our endoscopic unit from January 2007 to September 2010. After standardization of the EUS images, brightness values including the mean (T_mean_), indicative of echogenicity, and the standard deviation (T_SD_), indicative of heterogeneity, in the tumors were analyzed.

**Results:**

The T_mean_ and T_SD_ were significantly higher in GIST than in leiomyoma and schwannoma (p < 0.001). However, there was no significant difference in the T_mean_ or T_SD_ between benign and malignant GISTs. The sensitivity and specificity were almost optimized for differentiating GIST from leiomyoma or schwannoma when the critical values of T_mean_ and T_SD_ were 65 and 75, respectively. The presence of at least 1 of these 2 findings in a given tumor resulted in a sensitivity of 94%, specificity of 80%, positive predictive value of 94%, negative predictive value of 80%, and accuracy of 90.8% for predicting GIST.

**Conclusions:**

Digital image analysis provides objective information on EUS images; thus, it can be useful in diagnosing gastric mesenchymal tumors.

## Background

Mesenchymal tumor of the stomach is usually discovered incidentally during upper endoscopy for an unrelated condition, and is noted as a firm, protruding subepithelial lesion; however, larger tumors occasionally can cause bleeding [1]. Histopathologically, most of these tumors are completely or partially composed of spindle cells and display smooth muscle or nerve sheath differentiation. Most gastric mesenchymal tumors are gastrointestinal stromal tumors (GISTs) derived from the interstitial cells of Cajal [1-3]. GIST has a risk of metastatic relapse, especially in the peritoneum and liver, even after surgery for localized disease [4,5]. Therefore, all GISTs are considered potentially malignant and may require resection, even small intramural lesions of the stomach [5,6].

In practice, the differentiation of GISTs from benign gastric mesenchymal tumors, such as leiomyoma or schwannoma, is essential for effective clinical management. Endoscopic ultrasonography (EUS) is a valuable imaging tool for evaluating mesenchymal tumors because it enables the demonstration of a hypoechoic mass that is contiguous with the fourth hypoechoic layer of the normal gut wall [7-9]. Despite this fact, there are few studies on differentiation between GISTs and benign mesenchymal tumors with the use of EUS [9,10]. In addition, there are limitations in the analysis of the characteristic features of such tumors due to poor interobserver agreement as a result of subjective interpretation of EUS images [11,12].

Digital images consist of pixels (picture elements), which are the basic elements that compose a 2-dimensional picture. In digital image analysis, the distribution and spatial variation of pixels is computed using texture analysis in order to extract useful data. Recently, the usefulness of digital image analysis in distinguishing benign from malignant subepithelial lesions on EUS has been reported [13]. Therefore, the aim of this study was to evaluate the role of digital image analysis in distinguishing the features of GISTs from those of benign mesenchymal tumors on EUS.

## Methods

### Subjects

The medical records of all patients with histopathologically proven gastric GIST, leiomyoma, or schwannoma on surgically resected specimens who underwent EUS examination at our endoscopic unit from January 2007 to September 2010 were retrospectively reviewed. We enrolled 65 patients (27 men and 38 women) with a mean age of 55 years (range, 28–81 years), of which 50 had GIST, 6 leiomyoma, and 9 schwannoma. This study was reviewed and approved by the Institutional Review Board at Pusan National University Hospital.

### Histopathology

The tumors were histopathologically proved to be gastric mesenchymal tumors and were classified immunohistochemically as leiomyoma, schwannoma, or GIST [3]. Leiomyoma was defined as a desmin-positive and c-kit (CD117)-negative tumor, schwannoma as an S-100-positive and c-kit-negative tumor, and GIST as a c-kit-positive tumor. GISTs were divided into 4 groups in accordance with the consensus meeting report at the National Institutes of Health [6].

### Endoscopic ultrasonography

EUS was performed using a radial-scanning ultrasonic endoscope (GF-UM2000; Olympus, Tokyo, Japan) at 7.5 MHz, and all examinations were performed under intravenous conscious sedation (midazolam with or without meperidine). The tumor was scanned after filling the stomach with 400–600 mL of deaerated water. At least 10 still EUS images were obtained for each lesion, and these images were saved digitally in Windows bitmap format.

EUS images were reviewed by a single experienced endosonographer (G.H.K.) who was kept blinded to the final diagnosis. Only 1 still EUS image of the highest quality was selected for each lesion for further digital image analysis, which was performed on a standard desktop computer.

### Digital image analysis

**Figure 1:**
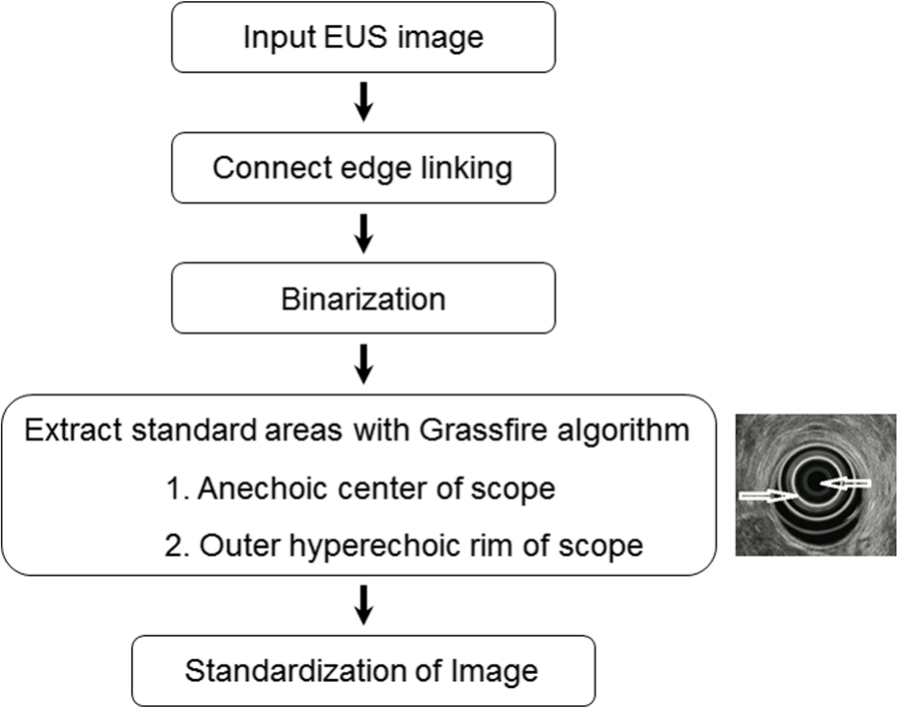
Flow diagram illustrating the standardization process of the EUS image.

EUS can display different image characteristics in accordance with various contrasts during a real examination. To minimize these differences, a standardization process was performed using the brightness values of the anechoic center and outer hyperechoic rim of the EUS scope, which have the least variability. Figure 1 shows the standardization process used in this study.

Image revision with histogram smoothing is also necessary to obtain better contrast because the original EUS image may be skewed by the brightness of the histogram and therefore may not be useful for analyzing the tumor area. Then, we applied an edge-linking method for all edge pixels repetitively in order to produce an edge. Edge-linking connected and recorded all values, satisfying formula (1) in a 3 × 3 area to current pixel.

(1) $$\left|\nabla G\left(x,y\right)-\nabla G\left(x',y'\right)\right|\leq \mathrm{Th}$$

**Figure 2:**
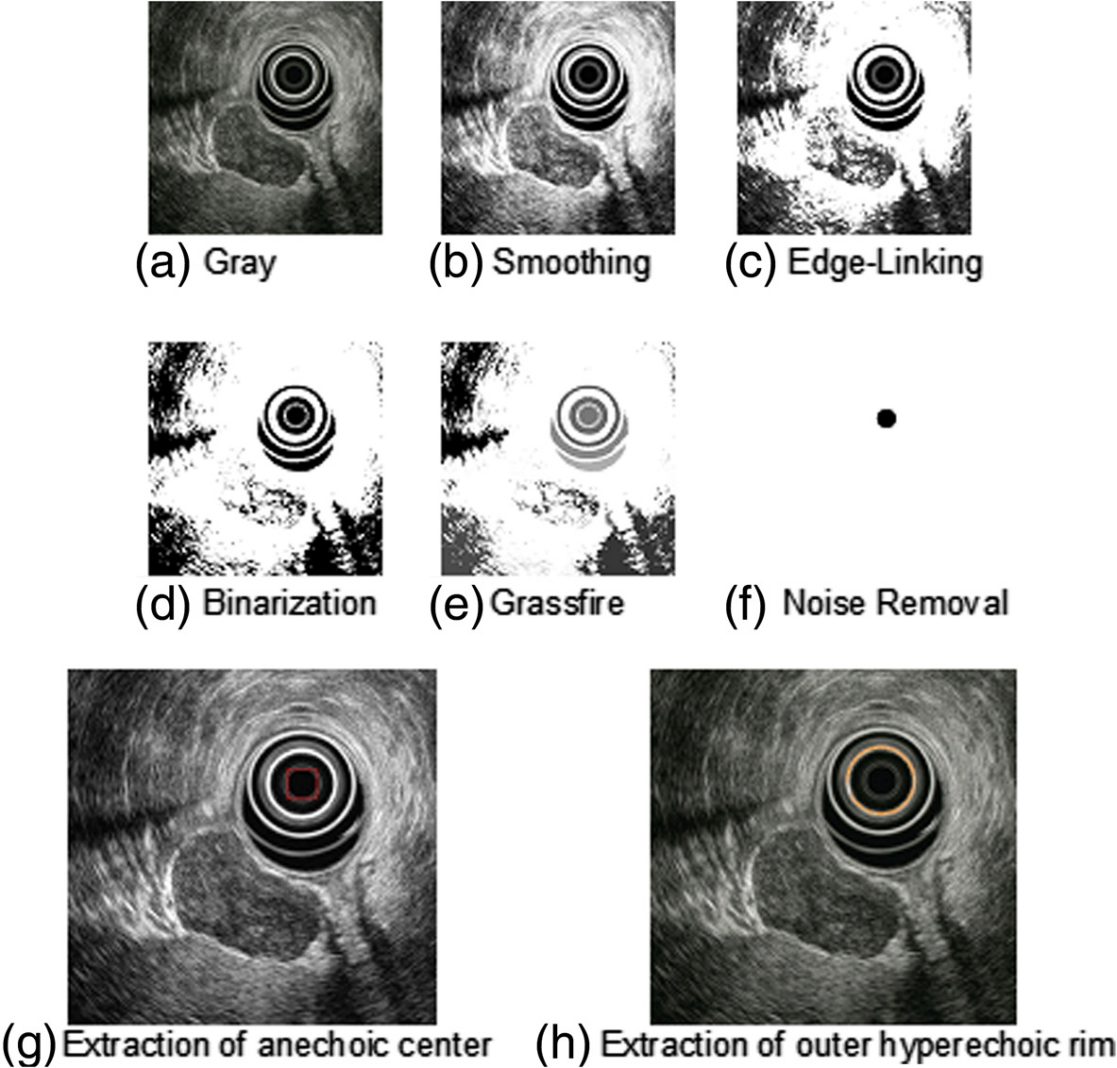
**Extraction process of the anechoic center and outer hyperechoic rim of the EUS scope. (a)** Gray image. **(b)** Smoothing method. **(c)** Edge-linking method. **(d)** Binarization. **(e)** Labeling with Grassfire algorithm. **(f)** Removal of noise using morphologic information. **(g)** Extraction of anechoic center of the scope. **(h)** Extraction of outer hyperechoic rim of the scope.

The threshold (*Th*) of formula (1) was set at 130, based on our preliminary study (data not shown). Then, the anechoic center of the EUS scope was extracted as an object with high-density pixels after applying binarization, labeling with Grassfire algorithm, and noise removal using morphologic information. The outer hyperechoic rim of the scope was extracted as the area that was brighter than the neighboring pixels, as shown in Figure 2.

Finally formula (2) was applied to complete the standardization process:

(2) $$\begin{array}{l} \mathrm{StandardGray}=\left(255-\mathrm{RimGray}\right)\times \frac{1+\left(255-\mathrm{RimGray}\right)}{\mathrm{CenterGray}}\\ \mathrm{If}\left(\mathrm{CenterGray}<X<\mathrm{StandardGray}\right),\mathrm{Then}\\ \;X=\frac{\mathrm{StandardGray}}{\mathrm{StandardGray}-\mathrm{CenterGray}}\times \left(X-\mathrm{StandardGray}\right)\\ \mathrm{Else}\;\mathrm{If}\;\left(\mathrm{StandardGray}<X<\mathrm{EdgeGray}\right),\;\mathrm{Then}\\ \;X=\mathrm{StandardGray}+\frac{255-\mathrm{StandardGray}}{\mathrm{RimGray}-\mathrm{StandardGray}}\\ \;\times \left(X-\mathrm{StandardGray}\right) \end{array}$$

where CenterGray and RimGray denote the brightness values of the anechoic center and outer hyperechoic rim of the scope, respectively, while StandardGray denotes a brightness value to differentiate the anechoic center from the outer hyperechoic rim.

**Figure 3:**
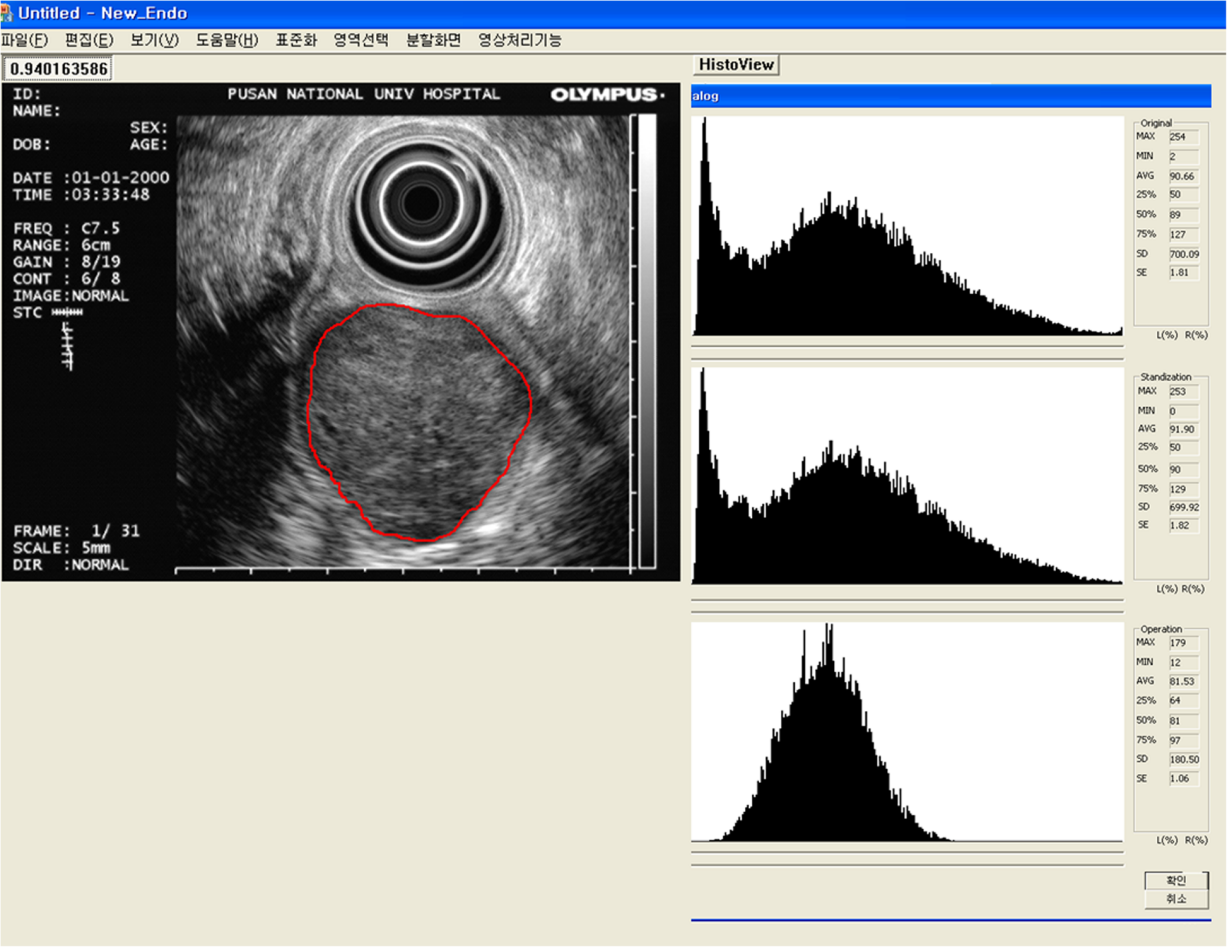
**An example of digital image analysis.** From the standardized image, a region of interest (ROI) is selected by an experienced endosonographer for tumor analysis. The final results for the ROI are expressed in the bottom histogram. The mean (T_mean_) and standard deviation (T_SD_) of the brightness values are 81.53 and 180.50, respectively.

From the standardized image, a region of interest (ROI) was selected by an experienced endosonographer (G.H.K.) for tumor analysis. The above method provides brightness information, including the minimum, maximum, mean (T_mean_), standard deviation (T_SD_), median, and interquartile values (Figure 3).

### Statistical analysis

All data are expressed as mean ± SD. The difference in T_mean_ and T_SD_ among the 3 groups (GIST, leiomyoma and schwannoma) was assessed using a one-way analysis of variance (ANOVA) test. A receiver operating characteristic (ROC) curve was applied to find the best sensitivity and specificity cut-off values of T_mean_ and T_SD_ for differentiating GIST from leiomyoma or schwannoma. Calculation of the sensitivity, specificity, positive and negative predictive values, and accuracy for differentiating GIST from leiomyoma or schwannoma was also performed. A p-value <0.05 was considered statistically significant. Statistical calculations were performed using SPSS version 12.0 for Windows software (SPSS Inc., Chicago, IL, USA).

## Results

In all EUS images, the T_mean_ and T_SD_ were calculated successfully after post-standardized image analysis. The T_mean_, which is indicative of echogenicity, was significantly higher in GIST than in leiomyoma and schwannoma (82.8 ± 22.5, 39.8 ± 18.9, and 47.0 ± 12.0, respectively; p < 0.001) (Table 1). In addition, the T_SD_, which is indicative of heterogeneity, was also significantly higher in GIST than in leiomyoma and schwannoma (83.5 ± 14.4, 54.3 ± 21.7, and 58.3 ± 17.5, respectively; p < 0.001). However, there was no significant difference in the T_mean_ or T_SD_ between leiomyoma and schwannoma.

**Table 1:** **Mean (T**_**mean**_**) and standard deviation (T**_**SD**_**) of the brightness values after digital image analysis of gastric mesenchymal tumors according to the histopathologic diagnosis**

	**GIST (n = 50)**	**Leiomyoma (n = 6)**	**Schwannoma (n = 9)**	**(*p- *** value)*
T_mean_ (mean ± SD)	82.8 ± 22.5	39.8 ± 18.9	47.0 ± 12.0	0.000
T^†^	a	b	b	
T_SD_ (mean ± SD)	83.5 ± 14.4	54.3 ± 21.7	58.3 ± 17.5	0.000
T^†^	a	b	b	

*GIST* gastrointestinal stromal tumor.

*Statistical significance was tested using one-way analysis of variance.

^†^The same letters indicate a non-significant difference between groups using Tukey’s multiple comparison test.

When the GISTs were classified into benign or malignant groups according to histologic risk classification, 31 cases were grouped as benign GISTs (very low risk, 7 cases; low risk, 24 cases) and 14 cases as malignant GISTs (intermediate risk, 10 cases; high risk, 4 cases). There was no difference in the T_mean_ or T_SD_ between benign and malignant GISTs (88.2 ± 21.7 vs 82.1 ± 23.0, p = 0.395; 86.9 ± 12.2 vs 83.3 ± 13.1, p = 0.373, respectively).

**Figure 4:**
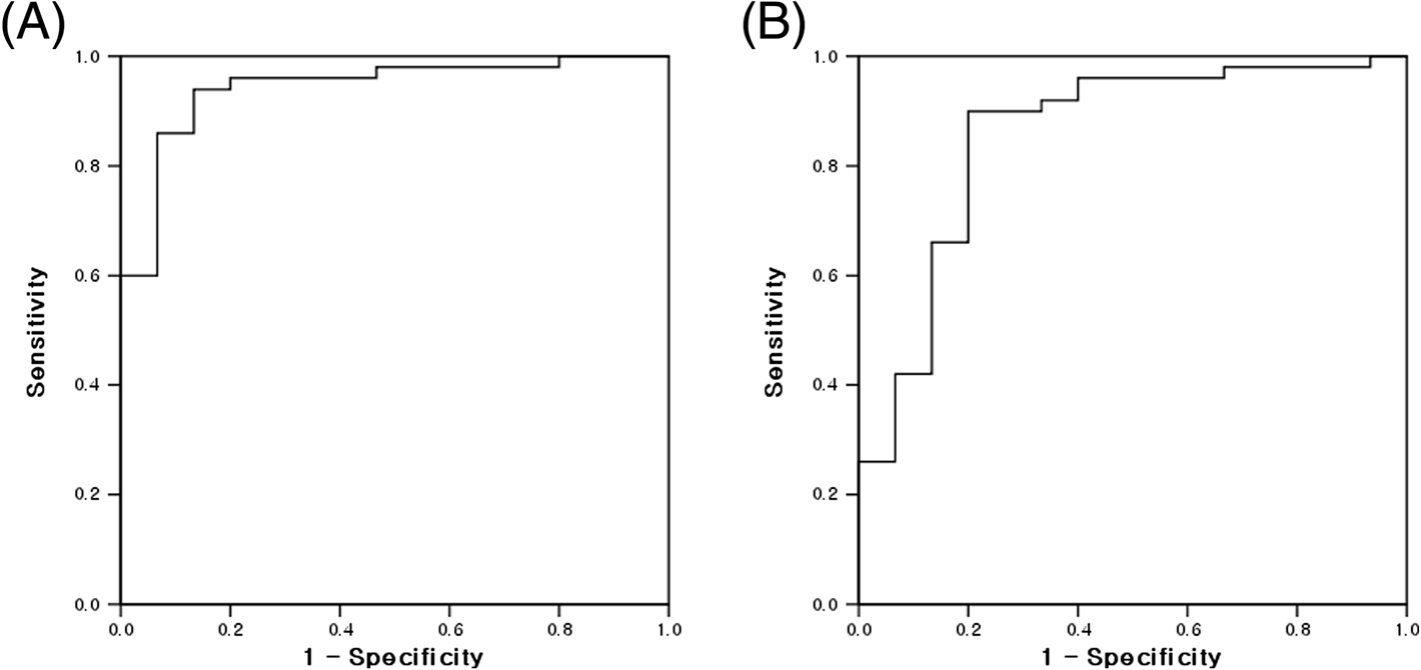
**Receiver operating characteristic (ROC) curve for differentiating gastrointestinal stromal tumor (GIST) from non-GIST mesenchymal tumors.** ROC curve of the **(A)** mean (T_mean_) and **(B)** standard deviation (T_SD_) of the brightness values that differentiate GIST from non-GIST mesenchymal tumors in the stomach.

An ROC curve was created to identify the best sensitivity and specificity cut-off values of T_mean_ and T_SD_ for differentiating GIST from leiomyoma or schwannoma (Figure 4). The sensitivity and specificity were almost optimized when the critical values of T_mean_ and T_SD_ were 65 and 75, respectively. Table 2 shows the values of T_mean_ ≥ 65 and T_SD_ ≥ 75 for predicting GIST. The presence of at least 1 of these 2 findings in a given tumor resulted in a sensitivity of 94%, specificity of 80%, positive predictive value of 94%, negative predictive value of 80%, and accuracy of 90.8% for predicting GIST.

**Table 2:** **Sensitivity, specificity, positive and negative predictive values, and accuracy of the mean (T**_**mean**_**) and standard deviation (T**_**SD**_**) of the brightness values that differentiate gastrointestinal stromal tumor (GIST) from non-GIST mesenchymal tumors in the stomach**

**Predicting GIST**	**Sensitivity, % (95% CI)**	**Specificity, % (95% CI)**	**PPV, % (95% CI)**	**NPV, % (95% CI)**	**Accuracy, % (95% CI)**
T_mean_ ≥ 65	86.0 (72.6-93.7)	93.3 (66.0-99.7)	97.7 (86.5-99.9)	66.7 (66.7-43.1)	87.7 (76.6-94.2)
T_SD_ ≥ 75	90.0 (77.4-96.2)	80.0 (51.4-94.7)	93.8 (81.8-98.4)	70.6 (44.0-88.6)	87.7 (76.6-94.2)
Of the above 2 features					
≥ 1	94.0 (82.5-98.4)	80.0 (51.4-94.7)	94.0 (82.5-98.4)	80.0 (51.4-94.7)	90.8 (80.3-96.2)
Both	82.0 (68.1-91.0)	93.3 (66.0-99.7)	97.6 (86.0-99.9)	61.0 (38.8-79.5)	84.6 (73.1-91.2)

*PPV* positive predictive value, *NPN* negative predictive value, *CI* confidence interval.

## Discussion

In our previous study, we evaluated the features that could differentiate GIST from leiomyoma on EUS; heterogeneity, hyperechogenic spots, a marginal halo, and higher echogenicity in comparison with the surrounding muscle layer were helpful for predicting GIST [9]. However, judgment of these findings on EUS images is subjective; this can result in poor interobserver agreement [11,12]. To overcome this limitation, we attempted to derive more objective findings from EUS images.

A EUS image is composed of pixels, and its echo density is expressed in brightness values from 0 (black) to 255 (white). Analysis of the brightness is, in principle, a method to evaluate the level of echogenicity (expressed as T_mean_) and the degree of homogeneity (expressed as T_SD_). In addition, EUS images can display different characteristics in accordance with various contrasts used during an examination. Therefore, to minimize these differences, we selected the brightness of the anechoic center and outer hyperechoic rim of the EUS scope, which have the least variability, and also standardized the EUS images.

After post-standardized image analysis, both the T_mean_ and T_SD_ were significantly higher in GIST than in leiomyoma and schwannoma. These results are consistent with those of previous studies that have reported higher echogenicity in comparison with the surrounding muscle layer, and heterogeneity is useful in diagnosing GIST [9,10,14]. In other words, we believe it is suitable to express some EUS findings as objective values after digital image analysis.

According to an ROC curve, the values of T_mean_ and T_SD_ showing the best sensitivity and specificity for GIST were 65 and 75, respectively. If either T_mean_ ≥ 65 or T_SD_ ≥ 75 was present, the sensitivity and specificity for predicting GIST were 94% and 80%, respectively, consistent with our previous results [9].

Next, we attempted to differentiate between benign and malignant GISTs on the basis of image analysis after dividing the GISTs into 2 groups (benign or malignant) according to histologic risk classification. However, we found no difference in the T_mean_ or T_SD_ between benign and malignant GISTs. Previous studies have suggested that large size, exogastric growth, ulceration, cystic changes, hyperechogenic foci, and irregularity of the margin favor a diagnosis of malignant gastrointestinal mesenchymal tumor [7,8,15,16]. In our previous report, only size was an independent predictor on multivariate logistic regression analysis [9]. Therefore, there is still a limitation in predicting the malignant potential of GIST with the use of image analysis.

This study has several limitations. First, this was a retrospective study that compared EUS features between GISTs and benign mesenchymal tumors using digital image analysis. Therefore, there might have been a potential bias when retrospectively reviewing the EUS images. During the EUS examination, we obtained at least 10 endosonographic images to determine the characteristics of gastric mesenchymal tumors; we hoped this would compensate, to some degree, for the limitation of this being a retrospective study. Second, although EUS examinations were performed, patients were selected for surgery according to the clinical opinions and decisions of the medical doctors. Third, the number of patients with leiomyoma or schwannoma included in this study was small, relative to the number of those with GIST. This limitation might be due to the fact that the most common mesenchymal tumor of the stomach is GIST and that other tumors, such as leiomyoma or schwannoma, are rarely encountered in clinics. Lastly, even though we analyzed only the EUS images obtained at 7.5 MHz in order to reduce differences between the images that could be due to different frequencies, the real settings of EUS, such as gain and contrast, were different in each case, which is a limitation inherent to a retrospective study. We did attempt to standardize the EUS images on the basis of the brightness values of the anechoic center and outer hyperechoic rim of the scope. However, this attempt to standardize the EUS images will not completely overcome the limitations of a retrospective study. Therefore, prospective studies will be needed that use the same conditions of settings such as frequency, gain, and contrast.

Gastric mesenchymal tumor is often asymptomatic, and is usually discovered incidentally during upper gastrointestinal endoscopy for an unrelated condition. The main problem in asymptomatic patients is to determine whether the tumor has a malignant potential. Because GISTs have malignant potential, gastric mesenchymal tumors should not be ignored, even if they are small, if the EUS features are suggestive of GIST. Therefore, if the digital image analysis suggests a high possibility of a GIST, it would be better to attempts to obtain tissue (such as by EUS-guided fine-needle aspiration or biopsy) or to resect the tumor (such as by endoscopic or surgical resection). Further large prospective studies are required to validate our results of EUS image analysis of gastric mesenchymal tumors.

## Conclusion

In conclusion, digital image analysis provides objective information on EUS images; thus, it can be useful in diagnosing gastric mesenchymal tumors. The results of EUS image analysis, such as T_mean_ ≥ 65 or T_SD_ ≥ 75, may help to differentiate GIST from leiomyoma or schwannoma.

## Consent

Written informed consent was obtained from the patient for the publication of this report and any accompanying images.

## Competing interest

The authors declare that they have no competing interests.

## Authors’ contributions

Study concept and design - GHK, GBK, and DYP; Acquisition of samples - GHK, DYP, and HKJ; Analysis and interpretation of data - GHK, SHL, TYJ, and DHK; Drafting of the manuscript - GHK and DYP; Statistical analysis - GHK and GAS; Obtained funding - DYP; Co-senior author and study supervision - GAS. All authors read and approved the final manuscript.

## Pre-publication history

The pre-publication history for this paper can be accessed here:

http://www.biomedcentral.com/1471-230X/14/7/prepub
